# Mural Variant of Unicystic Ameloblastoma in a Pediatric Patient: A Rare Case Report

**DOI:** 10.7759/cureus.11963

**Published:** 2020-12-07

**Authors:** Laavanya Marimuthu, Senthil Kumar, Vandana Shenoy, Mohammed Afradh, Ashwan Paranthaman

**Affiliations:** 1 Oral and Maxillofacial Surgery, Thai Moogambigai Dental College & Hospital, Chennai, IND

**Keywords:** ameloblastoma, unicystic ameloblastoma, mural ameloblastoma, enucleation, carnoy's solution

## Abstract

Unicystic ameloblastoma (UA) differs from conventional ameloblastoma by presenting in the younger population with a lower recurrence rate and has a unilocular appearance on radiograph mimicking dentigerous cysts. Among the three variants of UA, the mural variant has a tendency to recur. Here we present a case of mural variant of UA in a pediatric patient treated conservatively with enucleation followed by application of Carnoy’s solution to the cystic cavity. The aim of this case report is to emphasize conservative management in the treatment of UA in pediatric patients considering growth and development in children.

## Introduction

Ameloblastoma is the most common benign, odontogenic tumor of epithelial origin [1,2]. The WHO defines ameloblastoma as a benign but locally invasive polymorphic neoplasm consisting of proliferating odontogenic epithelium, usually having a follicular or plexiform pattern, lying in a fibrous stroma. It presents as an asymptomatic slow growing lesion which predominantly occurs in the mandible [2]. There is no sex predilection with a peak incidence in the third to fourth decade of life [1,3]. Ameloblastoma presents as three variants, namely the conventional (solid)/multicystic ameloblastoma, unicystic ameloblastoma (UA) and the peripheral (extraosseous) ameloblastoma [1,3]. Among the three variants, the conventional type is the most common variety and UA is a comparatively rare type [3]. UA differs considerably from the conventional type by presenting at a relatively younger age group, being typically unilocular on radiographs, appearing cystic macroscopically and also by responding better to conservative treatment modalities [4]. Though several treatments ranging from enucleation to resection are employed for the treatment of UA, a more conservative treatment is preferred for the pediatric population [5]. Here we present a case of UA in a 9 year old child treated conservatively by enucleation followed by application of Carnoy’s solution.

## Case presentation

**Figure 1:**
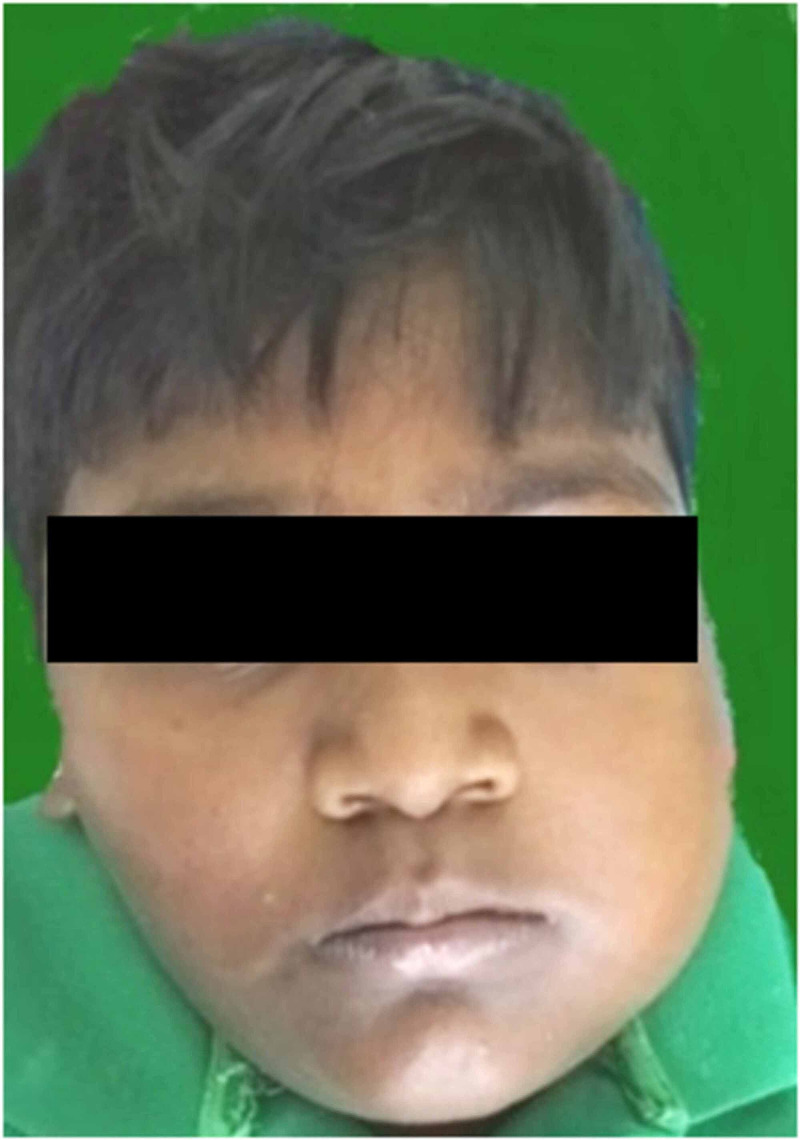
Frontal view showing diffuse swelling over the left maxillary and mandibular region

A nine year old male child presented to the department of oral and maxillofacial surgery with a chief complaint of swelling in the lower left side of the face for a period of two months. On examination, a diffuse swelling was present on the lower left side of the face with reduced mouth opening. The swelling was tender on palpation with a firm consistency (Figure 1). There was paresthesia on the left side of the lower lip. Intraoral examination revealed a diffuse swelling of size approximately 5cmx3cm in relation to the lower left first molar and second molar region, which was firm and tender on palpation. Egg shell crackling was felt. The mandibular left permanent first molar was mobile.

**Figure 2:**
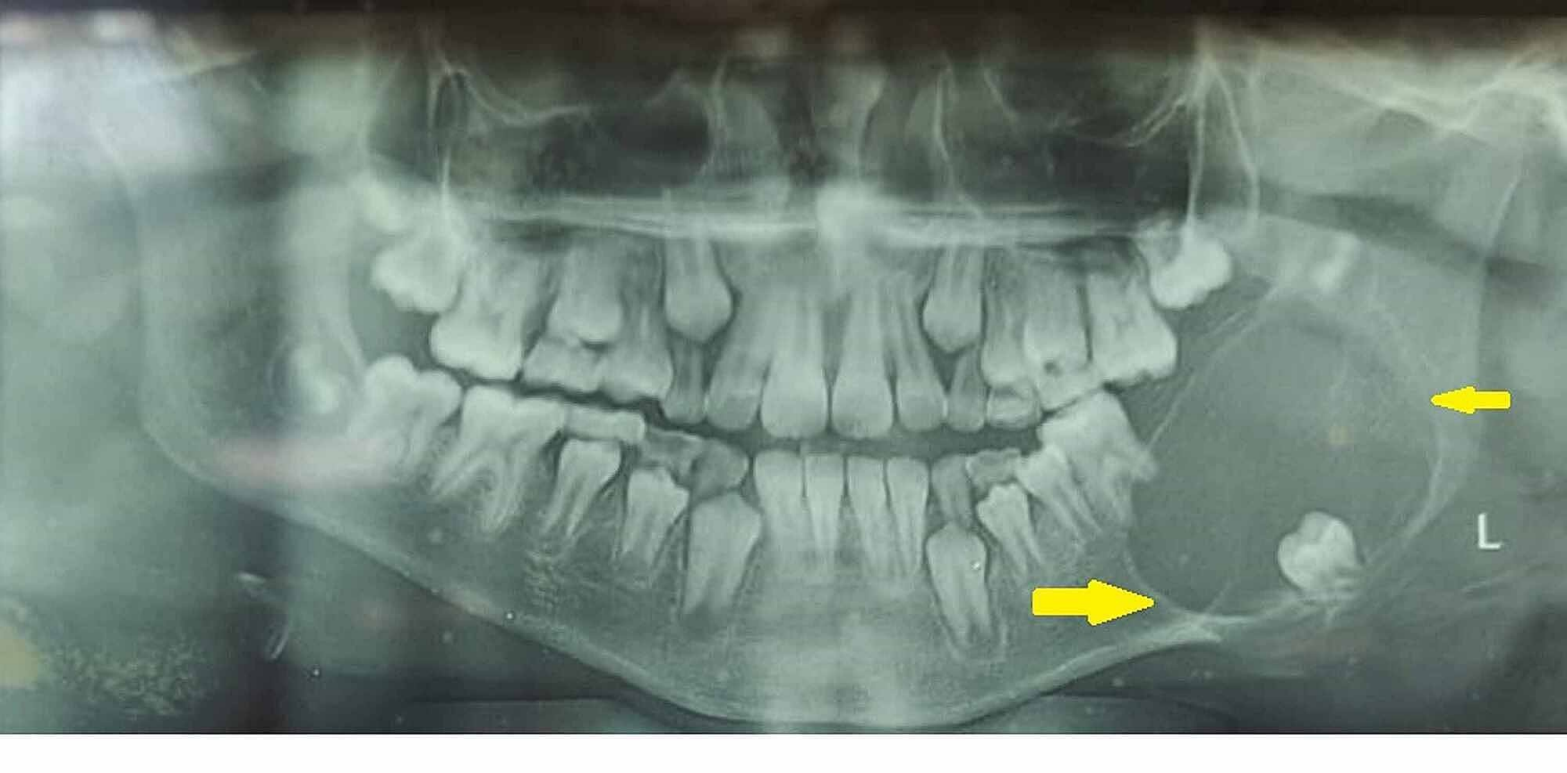
Orthopantamogram showing radiolucent lesion on left mandible with sclerotic borders denoted by yellow arrows

**Figure 3:**
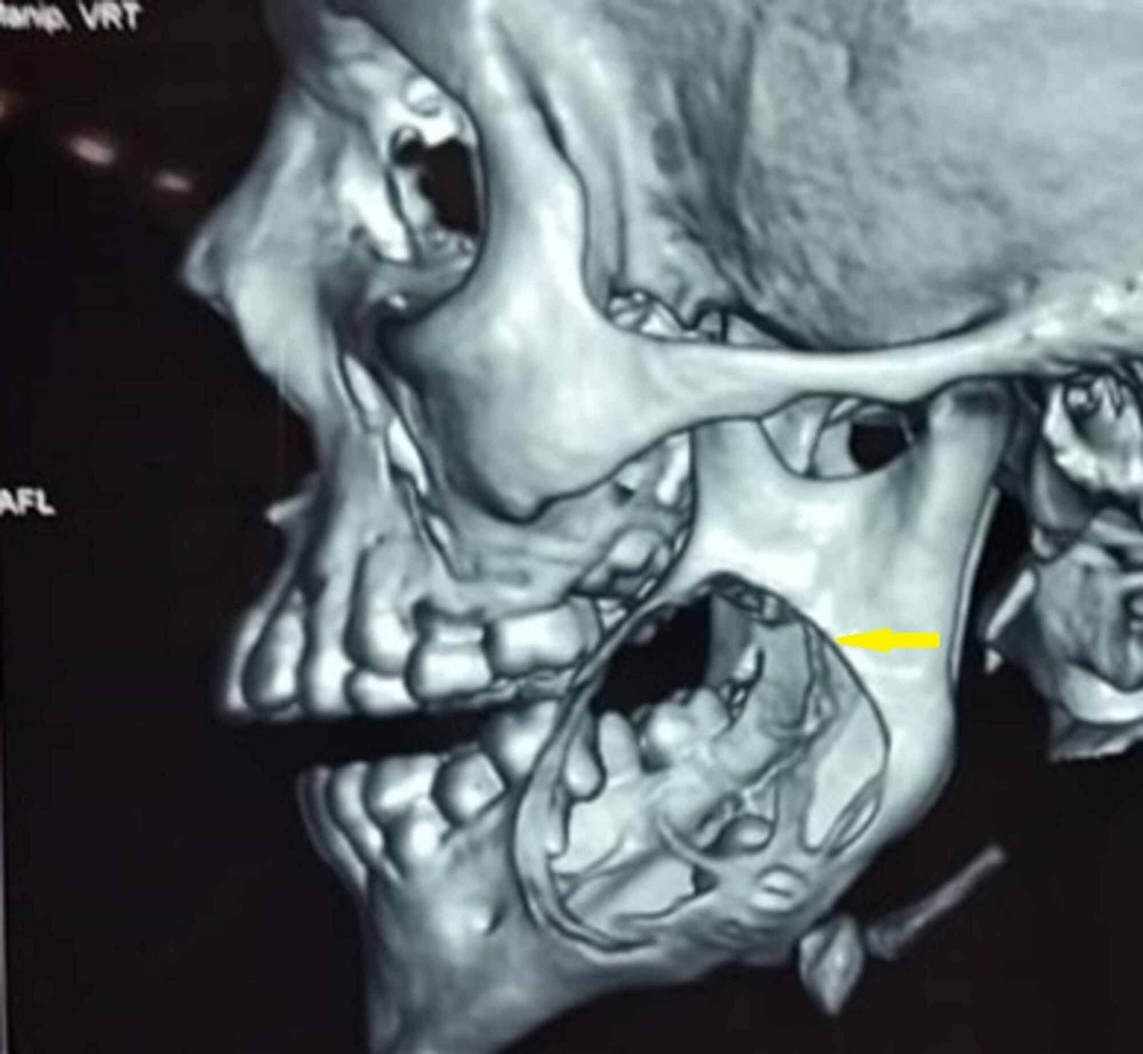
Computed Tomography 3D recontructed image showing the lesion occupying the left mandibular ramus

**Figure 4:**
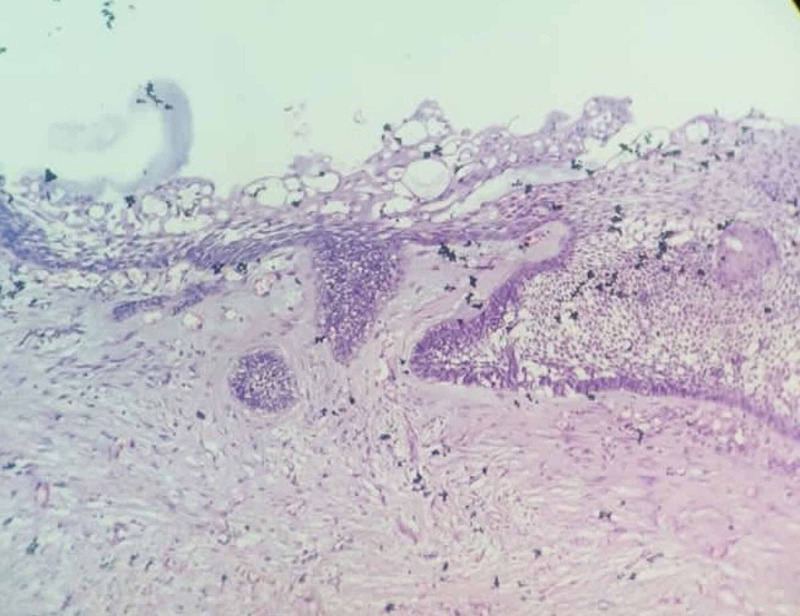
Hematoxylin & Eosin stain showing histopathological mural variant of UA under 10X magnification.

Orthopantamogram revealed unilocular radiolucency with sclerotic border on the left side involving the permanent molars with the second molar being pushed to the inferior border of the mandible and third molar displaced to the coronoid notch (Figure 2). CT scan revealed expansile lesion with thinning of both buccal and lingual cortex (Figure 3). A straw color fluid was aspirated from the cystic lesion. Based on the clinical diagnosis of dentigerous cyst, an incisional biopsy was performed. Histopathology revealed a luminal variant of UA (Figure 4).

**Figure 5:**
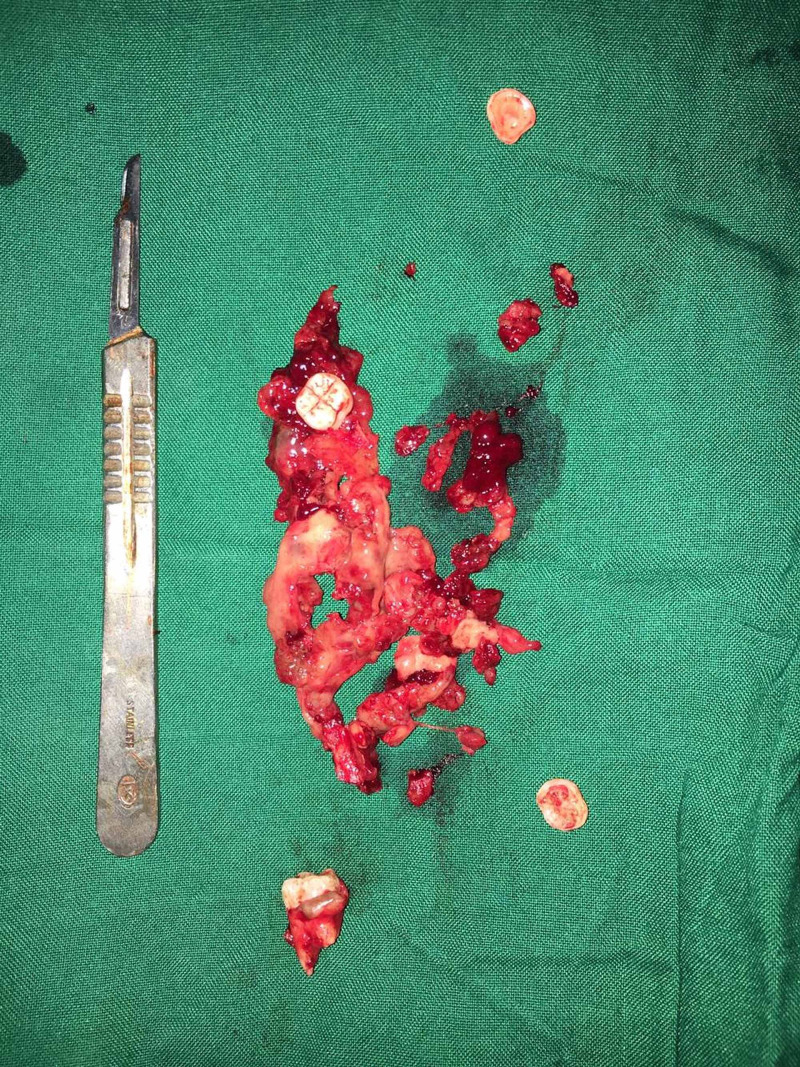
Excised specimen along with teeth

**Figure 6:**
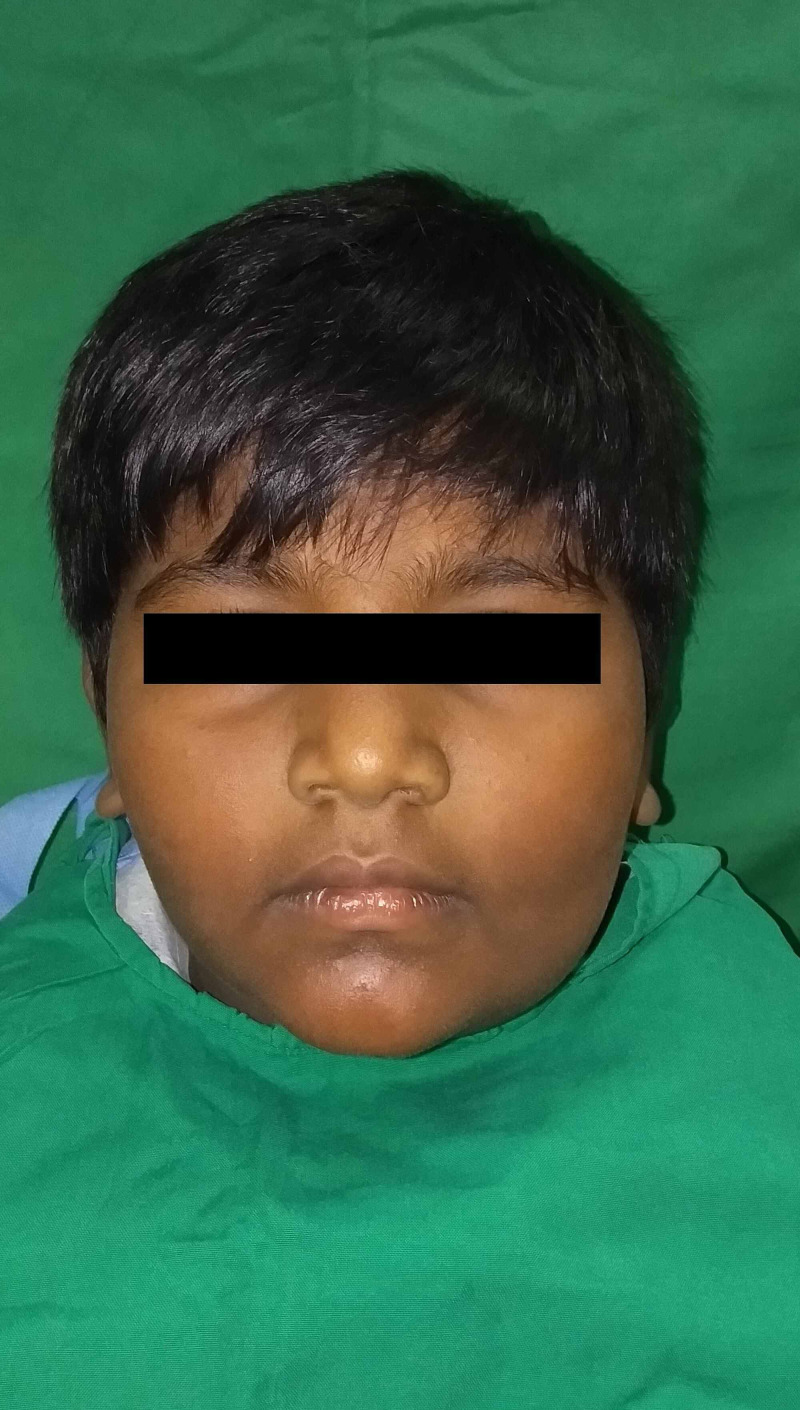
Frontal view of the patient at one and half year follow up period

**Figure 7:**
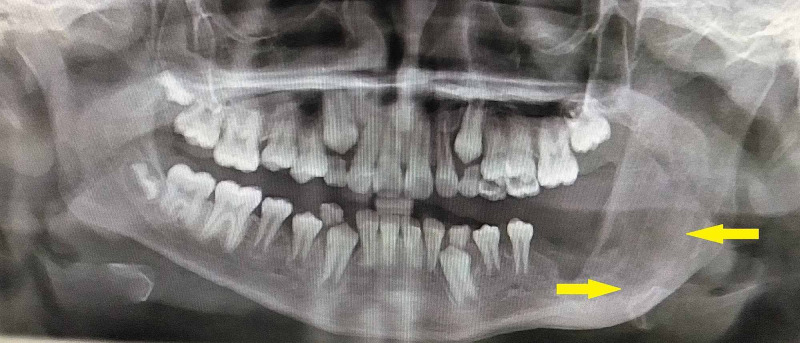
Orthopantamogram taken at one and half year follow up period showing bone formation (denoted by yellow arrows)

Though the lesion was extensive, a conservative treatment modality of enucleation was preferred considering the age of the patient. Under general anesthesia, the lesion was enucleated along with the involved permanent molars followed by application of Carnoy’s solution for a period of three minutes (Figure 5). Care was taken to prevent exposure of Carnoy’s solution to the inferior alveolar nerve. The cavity was packed with iodoform gauze for the wound to heal by secondary intention. Post operative recovery was satisfactory. Histopathologic evaluation of the excised specimen confirmed the lesion as mural variant of UA. The patient was followed for a period of one and half years. Orthopantamogram taken after a one and half year period showed areas of bone formation in the mandible (Figures 6,7).

## Discussion

Robinson and Martinez in 1977 defined UA as a cystic cavity lined by ameloblastic epithelium [6,7]. UA accounts for about 6% of ameloblastomas and occurs in younger age group with about 50% of the patients presenting in the second decade of life [3,7,8]. Clinical diagnosis of UA is challenging and it is frequently misdiagnosed since they show clinical and radiographic features similar to odontogenic cysts, most commonly a dentigerous cyst. Hence, histopathologic confirmation is mandatory to arrive at a final diagnosis [6,7]. Ackerman in 1988 classified UA on the basis of histopathologic features into three groups [7]:

Group I: Luminal UA where the tumor is confined to the luminal surface of the cyst

Group II : Intraluminal UA when there is nodular proliferation into the lumen without infiltration of tumor cells in the connective tissue wall.

Group III: Mural UA where there is presence of invasive islands of tumor cells in the connective tissue wall of the cyst

Our case falls under Group III as mural variant of UA. The mural variant is believed to show a behaviour similar to that of conventional solid ameloblastoma with a propensity for recurrence after enucleation because of the presence of tumor cells in the fibrous tissue capsule [9]. However, there is no clarification on whether the mural invasion can extend beyond the capsule to the adjacent bone [9].

The treatment of UA can be radical or conservative and this subject has always been controversial [10]. Radical treatment involves segmental or marginal resection of the lesion followed by placement of reconstruction plates whenever required. On the other hand, conservative treatment comprises of enucleation with or without curettage and marsupialization followed by enucleation [8]. Adjunctive therapy including thermal or chemical cauterization, cryotherapy and radiotherapy can be employed following primary treatment [6].

The effectiveness of a particular treatment modality is determined by means of the recurrence rate after treatment. The rate of recurrence is lesser with radical treatment when compared to a more conservative approach. The recurrence rate after treatment of UA ranges from 10%-25% [8]. Lau and Samman studied the recurrence rate in UA following various treatment modalities and observed the recurrence rate was 30.5% (highest) following enucleation alone, 18% for marsupialization, 16% for enucleation followed by application of Carnoy’s solution and 3.6% (lowest) for resection [6]. Though low recurrence rate is reported following resection, radical treatment is avoided in children for the following reasons (1) continuing facial growth in children and presence of a highly reactive periosteum (2) presence of unerupted permanent teeth (3) may cause disfigurement and masticatory issues which can be psychologically disturbing to the child. A conservative line of treatment plays an excellent role in pediatric and adolescent patients since it is associated with faster bone fill and restoration of normal bony architecture [4]. Since our case was a nine year old child, and the lesion was in mandible, we decided to opt for the more conservative treatment of enucleation followed by application of Carnoy’s solution.

Carnoy’s solution, first described by Culter and Zollingerin 1933, penetrates cancellous bone spaces consequently devitalizing and fixing the tumor cells [6]. The general procedure to use Carnoy’s solution in bone cavities is to apply the solution for a period of three to five minutes and then rinsing the cavity. Utmost care should be taken to prevent the contact of the solution to the inferior alveolar nerve [6]. In our case, we took adequate precautions to protect the inferior alveolar nerve.

On the basis of their study, Lee et al. suggested that the use of Carnoy’s solution after enucleation of UA with mural invasion could be adopted as a standard treatment protocol and they found that there was only 10% chances of recurrence following treatment of UA with enucleation followed by Carnoy’s solution application; among the patients who had recurrence, two patients were treated without extraction of the involved teeth which could be attributed to the fact that tumor remnants could have been possibly left around the tooth root apex [9]. In our case too, the involved teeth were removed to avoid this possibility.

Our case showed an excellent prognosis following conservative treatment with good bone formation within a period of one and half years justifying the avoidance of radical surgery in a young patient.

## Conclusions

The mural variant of UA has a high potential for recurrence when compared with other types of UA. Adult patients with mural UA can be treated by radical surgery to prevent recurrence. However, the same treatment protocol cannot be applied to the pediatric population since resection may cause an alteration in craniofacial development leading to functional and esthetic damage which can directly affect their quality of life. A more conservative approach of enucleating the lesion followed by application of Carnoy’s solution seems to be a promising treatment modality of UA in the pediatric age group. However, this warrants confirmation by future studies.
